# The Gastric Microbiome Is Perturbed in Advanced Gastric Adenocarcinoma Identified Through Shotgun Metagenomics

**DOI:** 10.3389/fcimb.2018.00433

**Published:** 2018-12-12

**Authors:** Yuan-Liang Hu, Wei Pang, Yun Huang, Yan Zhang, Chao-Jun Zhang

**Affiliations:** ^1^Graduate School, Third Military Medical University (Army Medical University), Chongqing, China; ^2^Department of General Surgery, Navy General Hospital, Beijing, China

**Keywords:** gastric adenocarcinoma, shotgun metagenomics, microbiome, inflammation, human

## Abstract

**Objective:** Dysbiosis of gastric microbiota such as *Helicobacter pylori* plays a significant role in pathogenesis and progression of gastric cancer. Our aim was to evaluate the composition and functional effects of gastric microbiota in superficial gastritis (SG) and advanced gastric adenocarcinoma (GC).

**Methods:** We carried out shotgun metagenomic sequencing on gastric wash samples from 6 patients with GC and 5 patients with SG. The taxonomic composition was profiled using MetaPhlAn2 and functional gene pathway was profiled using HUMAnN2. Differences in microbial composition and pathways between the two patient groups were assessed via LEfSe.

**Results:** The gastric microbiota in GC patients was characterized by reduced species richness, enrichment of 13 bacterial taxa and depletion of 31 taxa (*q* < 0.05). The most representative taxa which were abundant in GC corresponded to the commensals or opportunistic pathogens that usually colonize the oral cavity, including genera *Neisseria, Alloprevotella*, and *Aggregatibacter*, species *Streptococcus_mitis_oralis_pneumoniae* and strain *Porphyromonas_endodontalis.t_GCF_000174815*. Each of the three GC-associated genera could separate GC from SG completely. In particular, *Sphingobium yanoikuyae*, a bacterium capable of degrading carcinogenic compounds, was depleted in GC. Functionally, pathways associated with the biosynthesis of lipopolysaccharide (LPS) and L-arginine were enriched in GC, whereas pathways involved in the fermentation of short chain fatty acids (SCFAs) and branched amino acid metabolism were more abundant in SG.

**Conclusions:** Our results present new alterations in the gastric microbiome in patients with GC from a whole-genome perspective, suggesting that microbiome composition and function can be used for prognosis and diagnosis of GC.

## Introduction

Gastric cancer is the third leading cause of cancer-related deaths worldwide, with an estimated 1 million new cases recorded yearly, and half of these cases occurring in Eastern Asia (Torre et al., 2015). In China, many patients diagnosed with gastric cancer are usually in the advanced stages. Previous researches have established that genetic susceptibility and environmental factors including microbial infections contribute to carcinogenesis (Sears and Garrett, 2014; Chen J. et al., 2017). Moreover, cancer progression can be fueled by the interaction between microbiota and host immune system, especially in the human gastrointestinal tract where bacteria are abundant and the immune system is highly reactive (Gagliani et al., 2014). *Helicobacter pylori* (*H. pylori*) infection is the most important risk factor for gastric cancer (Polk and Peek, 2010). *H. pylori*-induced inflammation and injury can progressively destroy the architecture and function of the gastric epithelium (Amieva and Peek, 2016). However, successful eradication of *H. pylori* does not completely prevent the development of gastric carcinoma, and only ~1% of those infected develop gastric cancer (Ma et al., 2012; Lee et al., 2016; Shah, 2017). These statistics suggest that other factors might be involved in the carcinogenesis of gastric cancer and further investigation is required.

Two studies demonstrated that complex gastric microbiota can promote *H. pylori*-induced gastric cancer by hastening the onset of malignancy and promoting tumor progression using hypergastrinemic insulin-gastrin (INS-GAS) transgenic mouse model (Lofgren et al., 2011; Lertpiriyapong et al., 2014). Additionally, several studies, using high-throughput sequencing approaches to characterize the human gastric microbiota, have provided evidence that alterations of gastric microbiota other than *H. pylori* are associated with gastric cancer carcinogenesis (Yang et al., 2016; Castaño-Rodríguez et al., 2017; Coker et al., 2017; Ferreira et al., 2017; Yu et al., 2017; Hsieh et al., 2018). However, all these studies were based on genetic analysis of 16S rRNA marker which despite contributing to our understanding about microbiota in gastric carcinoma, limit our ability to characterize other key microbial genes that might be involved in this disease (Knight et al., 2017, 2018). Shotgun metagenomic sequencing provides higher-level of taxonomic and functional resolution by targeting the entire genomic content of a sample (Quince et al., 2017). Therefore, we performed shotgun metagenomics survey on the gastric microbial communities of 6 advanced gastric adenocarcinoma cases and 5 superficial gastritis controls to characterize the compositional and functional changes associated with gastric malignancy.

## Materials and Methods

### Subjects Recruitment and Sample Collection

Six individuals with GC and 5 with SG were recruited in Beijing, China. Typical meals in Beijing include steamed bread, noodles, dumplings, rice, pancakes, and meats, such as mutton and beef. Beijing residents also habitually consume kebab. Subjects with GC (mean age 60.5 ± 6.5 years; male-to-female ratio 5:1) were recruited during hospitalization at the Department of General Surgery of Navy General Hospital. The diagnosis of malignancy was based on pathologic analysis of tissue biopsies and further confirmation was done by postoperative pathological analysis. Subjects with SG (mean age 55.2 ± 5.6 years; male-to-female ratio 4:1) were recruited during their hospital visits to the out-patient unit of the same department with mild to moderate epigastric discomfort, and they were diagnosed with SG using esophagogastroscopy. All the subjects were recruited between July 2017 and February 2018. Exclusion criteria included the use of antibiotics within 6 months (Dethlefsen et al., 2008; Dethlefsen and Relman, 2011; Knight et al., 2018), receiving chemotherapy or radiotherapy prior to the collection of specimens, the use of proton pump inhibitors or other digestive system drugs within 4 weeks. The study was approved by the institutional review boards of the Navy General Hospital, and all subjects provided written informed consent for obtaining study specimens for this research.

In this study, gastric wash samples were collected, which are less difficult to obtain compared to tissues and are relatively non-invasive. Additionally, these samples harbors a combination of mucosal microbes and luminal communities, which have not been previously assessed in GC patients. Samples were obtained by endoscopy between 8.30 and 9.30 a.m. Briefly, subjects were required to fast for a minimum of 8 h before the esophagogastroscopy was performed. When the endoscope entered the stomach, 150 mL sterilized sodium chloride physiological solution was used to wash the whole stomach wall before sucking all liquids into a sterilized suction bottle. Samples were immediately put on ice, transported to the laboratory and aliquoted within 30 min. An aliquot was used immediately for DNA extraction and the rest were stored at −80°C for further processing. Samples were delinked and unidentified from their donors.

### Sample Pretreatment and DNA Extraction

Considering the occasionally large amount of (unwanted) host DNA along with other substances including food and cellular metabolites in the samples collected, we designed some pretreatment steps to reduce human cells and food residue in these samples. First, a 50-mL falcon tube containing the sample was turned upside down several times to thoroughly homogenize the sample. Then, the sample was centrifuged at 400 g and 4°C for 10 min to separate the microbes from human cells and food residue. The supernatant was then transferred to a new 50-mL falcon tube, followed by filtration through a 5-μm Supor 200 PES Membrane Disc Filter, which allows the bacteria to pass through but not eukaryotic cells enclosed by membranes. The filtrate was further filtered through a 0.2-μm filter to enrich microbes. The remaining filter membrane was cut into small pieces for DNA extraction as previously described (Jiang et al., 2015), except that we used column instead of AMPure XP beads to purify the genomic DNA.

### Shotgun Metagenomic Sequencing

We constructed barcoded, paired-end libraries with an insert size of ~300 bp for each sample using the NEB Next ultra DNA library prep kit for Illumina after shearing the extracted DNA by the S2 Focused-ultrasonicator. About 80 ng genomic DNA of each sample was used to prepare sequencing libraries. The libraries were then multiplexed and paired end (150 × 150 bp) sequencing was performed on the Illumina HiSeq X10 platform.

### Prevention of Contamination and Negative Controls

Efforts were made to avoid contamination during the sample collection and laboratory operation steps. The endoscope and its internal piping system were sterilized by standard washing and elimination system with 20 min extended. In addition, 200 mL sterilized sodium chloride physiological solution was also used to wash the internal piping system before sampling. The filter was installed in the filter holder before autoclaving. The scissors and forceps used in the sample pretreatment and DNA extraction steps were all sterilized before use. Surgical mask and sterile surgical gloves were used in all the steps. Negative-control experiments were conducted to assess the degree of potential contamination. For this purpose, 150 mL sterilized sodium chloride physiological solution (instead of actual sample) was sucked into a sterilized suction bottle, and the absorbed liquid was subjected to the same procedure as the actual sample. DNA extracted from the negative controls was verified to be negligible by Qubit® 3.0 Fluorometer (< 0.05 ng/μL for negative controls compared to 9.82 ng/μL for actual samples), and by performing 0.2% agarose gel electrophoresis on the amplified products of 16s rRNA gene V4 region after 30 cycles of amplification (no DNA band was observed in the negative controls compared to a bright band observed at ~290 bp for actual samples).

### Data Processing and Taxonomical Analysis

We followed the Microbiome Helper standard operating procedure to process the shotgun metagenomic data (Comeau et al., 2017), The FastQC tool was used to examine the metagenomic raw reads. KneadData, a helpful wrapper of both Trimmomatic (Bolger et al., 2014) and Bowtie2 (Langmead and Salzberg, 2012), was used to trim low-quality sequences (parameters: “SLIDINGWINDOW:4:20 MINLEN:50”) and to remove unwanted reads from human genomes and PhiX sequence (parameters: “–very-sensitive –dovetail”). The processed reads were further used to characterize the taxonomic profiles by MetaPhlan2, which used unique clade-specific markers to detect the taxonomic clades present in a microbiome sample and estimate their relative abundance (Segata et al., 2012b). Alpha diversity was estimated by the Shannon index and species richness. Differences in alpha diversity were assessed using the Student's *t*-test. Beta diversity between groups was assessed using Bray-Curtis dissimilarity and Jaccard Index and further visualized using principal coordinate analysis (PCoA) plots. Non-parametric permutational multivariate analysis of variance (PERMANOVA) was used to test sample clustering in beta diversity analysis with 10,000 replicate permutations, and this test was performed using the Adonis function in R package Vegan.

The differential abundance of taxa between groups were identified through linear discriminant analysis (LDA) effect size (LEfSe) algorithm (Segata et al., 2011). Only taxon with LDA score >2 at a *P* < 0.05 (Kruksal–Wallis test) was considered significantly enriched. The false discovery rate (Benjamini–Hochberg FDR, *q* < 0.05) method was used to adjust the *p*-values for multiple test correction.

### Functional Analysis

Gene family abundance, pathway abundance and pathway coverage of each sample were determined directly from processed reads using HUMAnN2 (version:0.11.1) with default parameters (Abubucker et al., 2012). HUMAnN2 utilizes the UniRef90 (Suzek et al., 2015), MetaCyc (Caspi et al., 2018) and MinPath (Ye and Doak, 2009) databases combined with MetaPhlAn2 and ChocoPhlAn pangenome databases to characterize the genes and pathways present in sequenced datasets. The nucleotide-level and translated searches are accelerated by running Bowtie2 and Diamond (Buchfink et al., 2014), respectively. HUMAnN2 generated three output files containing gene family abundance, pathway coverage and pathway abundance. We focused our analysis on the output of pathway abundance, which provided comprehensive quantitative insight into the functional aspects of a microbial community. Differentially abundant pathways between groups were identified through LEfSe. A difference was considered statistically significant if LDA score >2 and *p* < 0.05 (Kruksal–Wallis test) after multiple test correction by FDR adjustment.

## Results

### Quality Metrics of Shotgun Metagenomics Survey

In this study, we compared the gastric microbiome of patients with GC and SG by shotgun metagenomics analysis. A total of 11 subjects were included with matched age, sex and body mass index (BMI) between GC(*n* = 6) and SG(*n* = 5) groups (Table 1, Supplementary Table 1). In total, more than 329 million, 150 bp paired-end reads were obtained corresponding to a mean of 29.91 million reads per sample. After trimming and filtering, we obtained 7.8 million non-human high-quality reads per sample for further analysis. The low proportion of the remaining reads after quality filtering may be due to the dominance of human genomes, which routinely make up over 90% of the sequencing data from human saliva, nasal cavity, skin, and vaginal specimens (Marotz et al., 2018). We obtained the relative abundance of microbial communities in each sample using MetaPhlan2 (Segata et al., 2012b) (Supplementary Table 2). The majority of the mapped reads were attributed to bacteria (99.46 ± 0.94% in GC and 99.46 ± 0.51% (s.d.) in SG) and a smaller proportion corresponded to viruses (0.54 ± 0.94% in GC and 0.53 ± 0.51% in SG).

**Table 1:** Features of subjects.

**Feature**	**GC (*n* = 6)**	**SG (*n* = 5)**	***p*-value GC vs. SG**
Males (*n*)	5	4	1^⋆^
Age (years)	60.5 ± 6.5	55.2 ± 5.6	0.2012^∙;^
Type II diabetes (*n*)	1	1	1^⋆^
BMI (kg/m^2^)	24.57 ± 3.33	25.60 ± 1.68	0.2353^∙^

GC, advanced gastric adenocarcinoma; SG, superficial gastritis; BMI, body mass index; Data are mean ± SD,

⋆ Fisher's exact test,

∙ *Mann–Whitney U-test*.

### The Gastric Microbiota Profile Differs Between GC and SG

**Figure 1:**
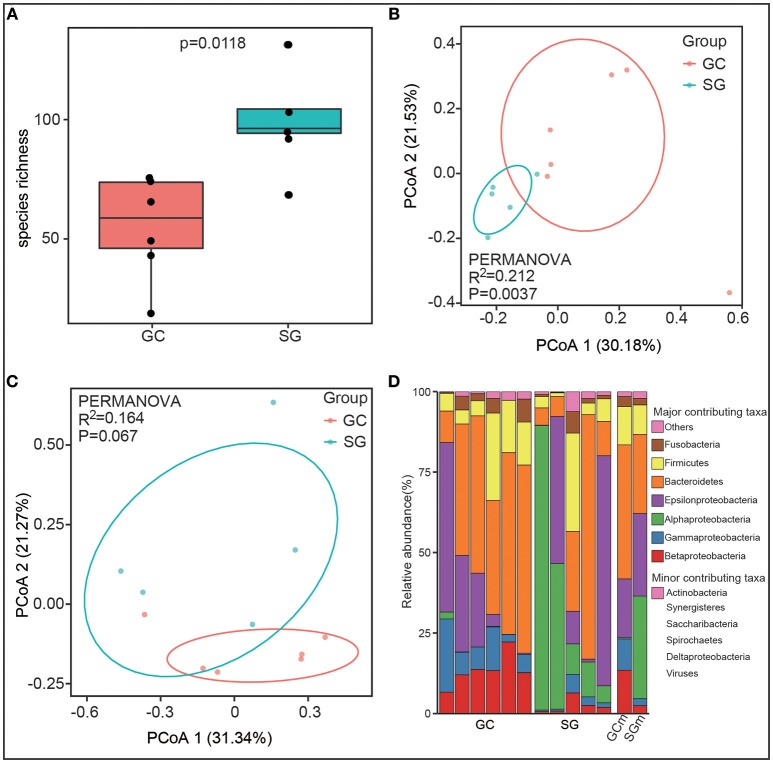
The gastric microbiota profile differs in GC and SG. **(A)** Species richness estimates (number of observed species), Student's *t*-test. Principal coordinate analysis (PCOA) plots of Jaccard index **(B)** and Bray-Curtis dissimilarities **(C)** in which samples were colored during clinical diagnosis. The percentage of diversity captured by each coordinate is shown. **(D)** Phyla distribution of the taxa in all samples. *Proteobacteria* was replaced by 5 class (*alpha, beta, delta, epsilon, gamma*). Biological replicates (6 replicates for GC and 5 replicates for SG samples) are displayed in separate stacked bars. The mean relative abundance of each phyla in each group is also shown in separate stacked bars. Major contributing phyla are shown in distinct colors and minor contributing taxa are grouped and displayed in pink. GC, advanced gastric adenocarcinoma; SG, superficial gastritis; GCm and SGm, the mean relative abundance of taxa in each group; PERMANOVA, non-parametric permutational multivariate analysis of variance.

Alpha diversity, the microbial diversity in each sample, was evaluated based on species richness and Shannon index. Species richness was highly dependent on clinical diagnosis (Student's *t*-test, *P* = 0.0118, Figure 1A) with higher richness values found in SG (102.2 ± 25.11) compared to GC (55.12 ± 19.72). For the Shannon index, which estimates richness and evenness of a community, was not significantly different between groups (Student's *t*-test, *P* = 0.46). This observation might suggest that the microbial communities in SG group were not evenly distributed, although more species were observed in this group.

To estimate the overall difference of microbial communities between groups with beta diversity, we evaluated dissimilarities between clinical diagnosis using Bray-Curtis and Jaccard index, which were further visualized in principal coordinate analysis (PCoA) plots. The total diversity captured by the top two principal coordinates was 52.61 and 51.71% for Bray-Curtis dissimilarity and Jaccard index, respectively. The gastric microbiome composition of patients with GC was significantly different from that of SG for Jaccard index (PERMANOVA, *R*^2^ = 0.212, *P* = 0.0037, Figure 1B), but not for Bray-Curtis dissimilarities (PERMANOVA, *R*^2^ = 0.164, *P* = 0.067, Figure 1C).

### Taxonomic Changes in GC Microbiome

Overall, the bacterial composition was dominated by phyla *Proteobacteria* (41.84% in GC and 62.13% in SG) and *Bacteroidetes* (41.64% in GC and 24.54% in SG), followed by *Firmicutes* (11.88% in GC and 9.18% in SG) and *Fusobacteria* (3.10% in GC and 2.01% in SG) in both groups (Figure 1D). At the class level, the microbiome of GC was characterized by significant enrichment of *Betaproteobacteria* (13.46 ± 4.59% in GC and 2.47 ± 2.08% in SG) and *Gammaproteobacteria* (9.71 ± 6.71% in GC and 2.18 ± 1.96% in SG), and depletion of *Alphaproteobacteria* (0.39 ± 0.77% in GC and 31.82 ± 31.7% in SG) compared to SG (Mann–Whitney *U*-test, Benjamini–Hochberg FDR, *q* < 0.05). *H. pylori*, which belongs to the class *Epsilonproteobacteria*, was not differently abundant between the groups based on clinical diagnosis (17.77 ± 19.16% in GC and 25.29 ± 28.49% in SG, Mann–Whitney *U*-test, *P* = 0.71, Figure 1D). These results showed that gastric microbial communities differ in GC and SG at high taxonomic levels, implying correlative changes may occur at lower taxonomic levels.

**Figure 2:**
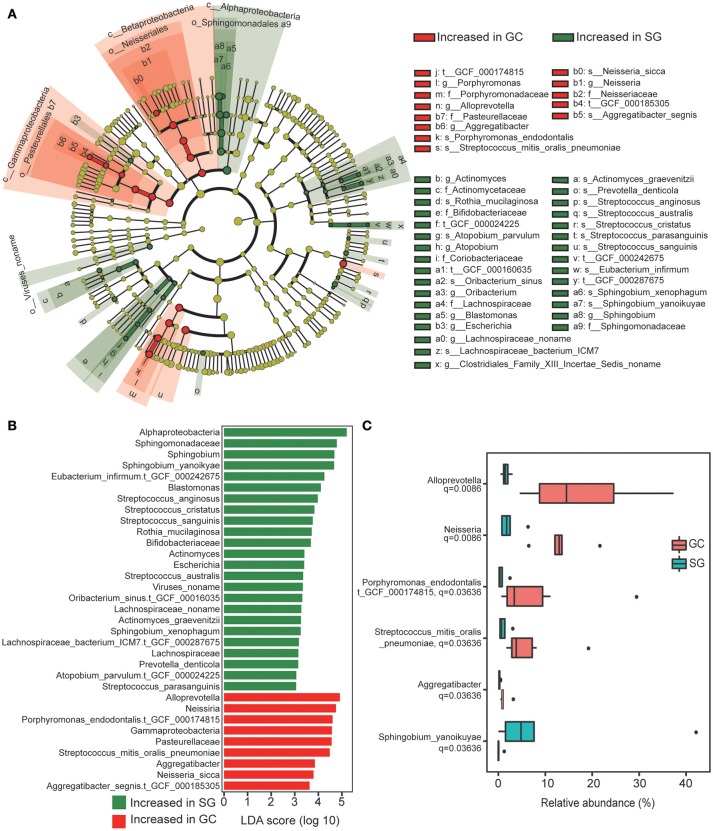
Taxonomic changes in GC microbiome. **(A)** Cladogram of the gastric microbial taxa associated with GC and SG. GC-enriched taxa are colored in red and SG-enriched taxa are in green. **(B)** Histogram of the linear discriminant analysis (LDA) scores for differentially abundant taxonomic features between GC and SG groups. Significance obtained by LDA effect size (LEfSe) at *p* < 0.05 (Kruksal–Wallis test) and LDAscore>2. **(C)** The six most representative taxa which were differentially abundant between GC and SG; Mann–Whitney *U*-test. *P*-values are adjusted by the false discovery rate (Benjamini–Hochberg FDR, *q* < 0.05) method; GC, advanced gastric adenocarcinoma; SG, superficial gastritis.

In total, 44 bacterial taxa were identified to be differentially abundant between the groups based on clinical diagnosis, including 8 families, 12 genera, 18 species and 6 strains (LDA score >2, Kruksal–Wallis test, Benjamini–Hochberg FDR, *q* < 0.05; Figures 2A,B; Supplementary Table 3). In GC group, enrichments in the taxa of *Betaproteobacteria* and *Gamaproteobacreria* were observed, including strain *Aggregatibacter_segnis.t_GCF_000185305*, species *Neisseria_sicca*, genus *Neisseria* and *Aggregatibacter* and family *Pasteurellaceae*. These taxa showed consistent enrichment across different taxonomic levels (Figure 2A). Additionally, strain *Porphyromonas_endodontalis.t_GCF_000174815*, species *Streptococcus_mitis_oralis_pneumoniae* and genus *Alloprevotella* were also highly abundant in GC. Enrichment in taxa of *Alphaproteobacteria* was observed in SG, including species *Sphingobium_xenophagum* and *Sphingobium_yanoikuyae*, genera *Blastomonas* and *Sphingobium* and family *Sphingomonadaceae*, these taxa also showed consistent enrichment across different taxonomic levels. Considering that some bacterial taxa shared the same clade and their relative abundance were identical, the taxa with lowest taxonomic level was chosen to represent these taxa for further analysis (Figure 2B). Interestingly, we found that all the taxa significantly enriched in GC were commensals or opportunistic pathogens which colonize in the oral cavity (Charlson et al., 2011), whereas the enriched taxa in SG included many commensals from gut with rather low relative abundance (Lozupone et al., 2012; Segata et al., 2012a).

We further screened for the most representative taxa which were differentially abundant between groups based on their relative abundance and prevalence. Those with average relative abundance >0.2% and prevalent in all the subjects of the target group were considered as the most representative. We finally obtained 5 GC-enriched taxa (*Neisseria, Alloprevotella, Aggregatibacter, Porphy-romonas_endodontalis.t_GCF_000174815, Streptococcus_mitis_ oralis_pneumoniae*) and one SG-enriched taxon (*Sphingobium_yanoikuyae*). Notably, we observed complete separation of the relative abundance of *Neisseria, Alloprevotella*, and *Aggregatibacter* between the groups (Figure 2C).

Taken together, these data showed that bacterial taxa were differentially abundant between the two groups, and the stomachs of GC patients were invaded by numerous mucosa-related bacteria which usually colonize the oral cavity as commensals or opportunistic pathogens.

### Functional Alterations of GC Microbiome

**Figure 3:**
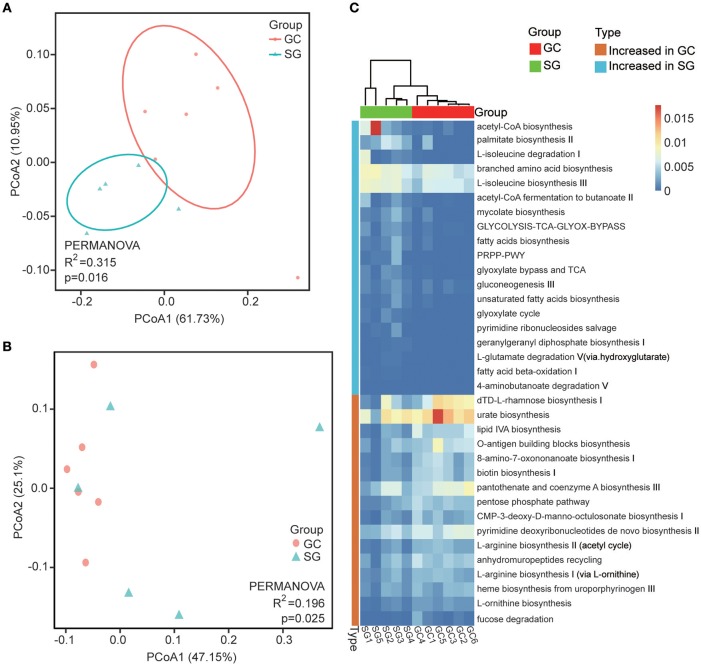
Functional alterations in GC microbiome. Beta diversity of functional gene pathways. Principal coordinate analysis (PCOA) plots of Jaccard index **(A)** and Bray-Curtis dissimilarities **(B)** in which samples were colored during clinical diagnosis. The percentage of diversity captured by each coordinate is shown. **(C)** Heatmap of differentially abundant functional gene pathways between GC and SG; Colored by the relative abundance of each pathway. Identified by LEfSe at *p* < 0.05 (Kruksal–Wallis test) and LDAscore>2. *P*-values are adjusted by the false discovery rate (Benjamini–Hochberg FDR, *q* < 0.05) method. GC, advanced gastric adenocarcinoma; SG, superficial gastritis; PERMANOVA, non-parametric permutational multivariate analysis of variance.

Overall, 349 organism-specific pathways were identified by HUMAnN2 based on the MetaCyc (Caspi et al., 2018) database (Supplementary Table 4). The majority of these pathways were associated with bacteria, which is in line with our taxonomic results generated by MetaPhlAn2. The overall differences among pathways between groups were evaluated by Bray–Curtis dissimilarity and Jaccard index, and were visualized in PCoA plots. The total diversity identified in the top two principal coordinates was 72.25 and 72.66% for Bray-Curtis dissimilarity and Jaccard index, respectively. Significant differences between clinical diagnoses were observed (PERMANOVA; *R*^2^ = 0.315, *P* = 0.016 for Jaccard index and *R*^2^ = 0.198, *P* = 0.025 for Bray–Curtis dissimilarity; Figures 3A,B).

In total, we identified 34 bacteria-specific metabolic pathways which were differentially abundant in the two patient groups through LEfSe (LDA score >2, Kruksal-Wallis test, Benjamini–Hochberg FDR, *q* < 0.05; Figure 3C; Supplementary Table 5). The enrichment of pathways associated with biosynthesis of LPS, biotin, heme, L-arginine and L-ornithine were observed in GC, while pathways associated with fermentation of SCFAs, branched amino acid metabolism, fatty acids biosynthesis and oxidation were more abundant in SG samples. Additionally, the enrichments of pathways associated with acetyl-CoA biosynthesis, glycolysis and tricarboxylic acid (TCA) cycle were observed in SG, but pentose phosphate pathways were preferentially abundant in GC samples. These results showed that the pathways involved in generating precursor metabolites and energy were significantly different between the two groups, and so were the pathways involved in amino acids biosynthesis and nucleotides metabolism.

## Discussion

The dysbiosis of human microbiota have been linked to a wide range of diseases including cancer, while inversely, some human microbes could enhance the efficacy of cancer immunotherapy and chemotherapy (Lynch and Pedersen, 2016; Roy and Trinchieri, 2017). However, unlike *H. pylori*, the roles of other gastric microbiota in GC are largely unknown. Moreover, there are currently no studies using shotgun metagenomic approach to survey the microbial communities in human stomach in terms of gastric malignancy. In this work, we profiled the gastric microbiota associated with GC and SG using shotgun metagenomic survey. We observed significant differences in the composition and function of gastric microbiota between GC and SG. The microbiome of GC was characterized by enrichment of several bacterial genera and species, which usually colonize the oral cavity as commensals or opportunistic pathogens. On the other hand, the microbiome of SG was characterized by enrichment of taxa corresponding to commensals from gut with a relatively low abundance.

In our study, the microbial richness (evaluated by species observed) but not the diversity (evaluated by Shannon Index), was found to be significantly higher in SG than GC. This observation was not completely consistent with previous studies using 16S rRNA marker gene analysis on gastric mucosa samples (Castaño-Rodríguez et al., 2017; Coker et al., 2017; Ferreira et al., 2017; Li et al., 2017). In fact, there are some discrepancies across these studies in terms of alpha diversity of gastric microbiota. There are many factors that may contribute to these discrepancies, including the physiological status of the host, sample type, experimental method, and bioinformatic approach (Sinha et al., 2017). To overcome some of these factors, we used a uniform method to collect and process the gastric wash samples obtained from two patient groups with matched age, gender and BMI, then performed bioinformatic analysis using widely accepted tools for shotgun metagenomics survey (Segata et al., 2011, 2012b; Abubucker et al., 2012; Comeau et al., 2017).

We observed consistent alterations of gastric microbiota communities across different taxonomic levels both in GC and SG. At the phylum level, *Proteobacteria, Bacteroidetes, Firmicutes*, and *Fusobacteria* were most abundant in both groups, which is in line with previous studies (Bik et al., 2006; von Rosenvinge et al., 2013; Nardone and Compare, 2015; Ferreira et al., 2017). From our findings, the relative abundance and prevalence of *H. pylori* were not significantly different between the two groups. This result differed with those from previous study where *H. pylori* decreased in gastric carcinoma compared to chronic gastritis (Ferreira et al., 2017). Despite this, we observed enrichment of *Betaproteobacteria* and *Gammaproteobacteria* in GC. These findings reveal that the none-*H. pylori Proteobacteria* may be closely correlated with GC.

Interestingly, the most representative taxa identified to be significantly abundant in GC were known members of commensals or opportunistic pathogens that usually colonize the oral cavity, including *Neisseria, Alloprevotella, Aggregatibacter, Porphyromonas_endodontalis.t_GCF_000174815*, and *Streptococcus_mitis_oralis_pneumoniae*. Notably, the relative abundance of genera *Neisseria, Alloprevotella* and *Aggregatibacter* can completely separate GC from SG, suggesting that they can be used for differential diagnosis of disease stages. However, there is need for further investigations using large cohorts. Extensive research has shown that oral microbiome are closely linked to gastrointestinal cancer (Ahn et al., 2012; Chen J. et al., 2017; Gao et al., 2018). Indeed, our findings are in agreement with a prospective nested case-control study that investigated the oral microbiome of patients with esophageal adenocarcinoma (EAC) and esophageal squamous cell carcinoma (ESCC) using 16S rRNA marker gene analysis (Peters et al., 2017). They demonstrated that depletion of genera *Neisseria* and species *Streptococcus pneumoniae* were associated with lower EAC risk. *Porphyromonas gingivalis*, another oral pathogen similar to *Porphyromonas endodontalis*, showed positive correlation with higher risk of ESCC. Moreover, another case-control study showed that *Porphyromonas gingivalis* and *Aggregatibacter actinomycetemcomitans*, two common oral pathogens, are risk factors for pancreatic cancer (Fan et al., 2018). Genus *Alloprevotella* was also found to be abundant in oral squamous cell carcinoma samples than in matched non-malignant samples (Zhao et al., 2017). Data from several studies showed significant enrichment of oral-associated bacteria in gastric carcinoma using 16S rRNA marker gene analysis, although the exact bacterial taxa were different from our findings (Castaño-Rodríguez et al., 2017; Coker et al., 2017; Yu et al., 2017). It should be pointed out that there are few studies directly investigating the oral microbiome of gastric carcinoma patients. A recent study reported that some oral pathogens, such as *Prevotella* and *Aggregatibacter*, were more abundant in the oral cavity of patients with gastric cancer compared with that of health controls (Sun et al., 2018). Further, oral health conditions and tooth flossing have been reported to be associated with gastric precancerous lesions (Salazar et al., 2012), and tooth loss was found to increase the risk of gastric non-cardia carcinoma (Abnet et al., 2001, 2005). These findings, in addition to the observations of our study show the potential role of oral-related bacterial commensals or pathogens in the pathogenesis and progression of gastrointestinal malignancy, which warrant further causal investigations. Importantly, the five bacteria taxa identified to be positively associated with gastric adenocarcinoma were not reported previously. Thus, our findings expand the current knowledge about their roles in gastrointestinal cancer from a whole genome perspective. Furthermore, the co-enrichment of these taxa in gastric adenocarcinoma indicates that a synergistic microbial network may exist thereby contributing to disease progress.

Contrary to the findings in our study, several studies using 16S rRNA marker gene analysis on gastric biopsy samples observed significant depletion of genera *Neisseria* in gastric carcinoma (Aviles-Jimenez et al., 2014; Ferreira et al., 2017). This discrepancy may be due to the differences in subject population, sample type and research method. Obviously, there are distinct differences between 16S rRNA marker gene analysis and shotgun metagenomics (Knight et al., 2018). With respect to sample type, we collected gastric wash samples instead of gastric mucosal specimens on the account of the following points. Several reports have shown that microbial communities of different anatomical gastric positions are similar (Bik et al., 2006; Coker et al., 2017; Yu et al., 2017), making it reasonable to study the gastric microbiota as a whole. Additionally, in addition to the washing step, the stomach fluid microbiota represents the union of mucosal microbes and luminal communities, which have not been previously assessed in GC patients. Since stomach fluid sampling is relatively non-invasive and has integrated properties, it has been used to characterize the gastric microbiota in various researches (von Rosenvinge et al., 2013; Al-Momani et al., 2016; Brawner et al., 2017). Therefore, a well-designed comparative analysis evaluating different methods could provide a deeper understanding on the observed discrepancy.

We observed significant enrichment of species *Sphingobium yanoikuyae* and genus *Sphingobium* in superficial gastritis with high relative abundance and prevalence. These taxa are capable of degrading xenobiotic compounds, particularly aromatic hydrocarbons, which are a group of organic pollutants with toxic, genotoxic, mutagenic and/or carcinogenic properties (Liu et al., 2012; Enguita and Leitão, 2013; Ghosal et al., 2016). These bacterial taxa have been reported to inhabit various niches in the human body, including female reproductive tract, bile, gut, and meconium (Goedert et al., 2014; Chen C. et al., 2017; Rogers et al., 2017; Stinson et al., 2018), suggesting their important roles in human health. In line with our results, *Sphingomonadaceae* has been reported to be more prevalent in the gastric biopsies of individuals inhabiting organs with lower risk of gastric cancer compared to other organs with high risk of cancer (Yang et al., 2016). Additionally, *Sphingobium yanoikuyae* has been reported to be negatively associated with breast cancer (Xuan et al., 2014; Chan et al., 2016). In line with these observations, our study demonstrates, for the first time, that bacteria corresponding to family *Sphingomonadaceae*, especially genus *Sphingobium* and species *Sphingobium yanoikuyae*, are negatively associated with advanced gastric adenocarcinoma.

To corroborate the microbiome dysbiosis observed in taxonomic composition, the disturbance of functional gene pathways was also analyzed in GC. Functional gene pathways of human microbiome in specific niches were found to be more constant and evenly diversified than taxonomic composition. In our study, the difference in beta diversity of functional pathways between the two groups of patients was more remarkable than that of taxonomic composition, indicating that GC microbiota with specific metabolic functions can efficiently colonize this specific niche. Studies have shown that LPS, a well-characterized toll-like receptor microbial ligand, increases inflammation in the tumor microenvironment and drives tumorigenesis (Rakoff-Nahoum and Medzhitov, 2009; Gagliani et al., 2014). Therefore, our observation that the enrichment of LPS biosynthesis pathways (lipid IVA biosynthesis and O-antigen building blocks biosynthesis) were enriched in GC implies that there is enhanced inflammation induced by GC microbiota. Interestingly, we observed remarkable differences in amino acid metabolism preferences between the microbiota of GC and SG. Pathways involved in branched amino acid metabolism were more abundant in SG, while biosynthesis of L-arginine and L-ornithine were enriched in GC, but the mechanism underling this difference and its impact on human health needs further investigation. SCFAs are the products of dietary fiber fermentation by intestinal microbiota, including acetate, propionate and butyrate. They play a role in maintaining microbiota homeostasis and integrity of the intestinal barrier, the suppression of inflammation and cancer (Louis et al., 2014; Yang and Yu, 2018). Consistent with these findings, we observed depletion of pathways (acetyl-CoA fermentation to butanoate II, L-glutamate degradation V and 4-aminobutanoate degradation V) associated with SCFAs production in GC, which indicates the existence of a more inflammatory microenvironment and dysbiotic microbial communities in GC. Contrary to our results, two studies profiling the microbiome of gastric mucosal samples using 16s rRNA marker gene survey observed the enrichment of pathways associated with the production of SCFAs in gastric cancer patients (Castaño-Rodríguez et al., 2017; Coker et al., 2017). This discrepancy could be due to the differences in subject population, sample type and research method. Further metagenomic, metatranscriptomic and metaproteomic surveys are required to confirm these results.

It should be noted that our study is limited by a relatively small sample size and therefore further longitudinal studies with larger numbers of subjects are needed. Moreover, the causal links between these candidate microbiota and GC susceptibility also need extensive investigation.

In conclusion, we conducted a shotgun metagenomics survey on the gastric microbiome of GC patients for the first time. We identified new GC-related bacteria and functional gene pathways, which extends the current knowledge about the role of gastric microbiota in GC. Further well-designed studies integrating microbial communities from the whole digestive tract are expected to provide a more comprehensive and detailed perspective on gastric microbiome. This will provide knowledge that can be exploited to develop strategies for prevention, early diagnosis and treatment of gastric cancer.

## Ethical Approval

This study was carried out in accordance with the recommendations of Human Specimen Study guidelines of the Institutional Review Board of Navy General Hospital of PLA. The protocol was approved by the Institutional Review Board of Navy General Hospital of PLA. All subjects gave written informed consent in accordance with the Declaration of Helsinki.

## Data Availability

Raw metagenomic reads supporting the conclusions of this article were uploaded to the NCBI Sequence Read Archive (SRA accession: PRJNA490628, https://www.ncbi.nlm.nih.gov/sra/PRJNA490628).

## Author Contributions

Y-LH-study concept and design, study conduct, analysis, and interpretation of data, manuscript preparation, critical revision of manuscript, approval of final draft submitted. WP, YH, and YZ-study conduct, critical revision of manuscript, approval of final draft submitted. C-JZ-study concept and design, study conduct, analysis and interpretation of data, manuscript preparation, critical revision of manuscript, approval of final draft submitted.

### Conflict of Interest Statement

The authors declare that the research was conducted in the absence of any commercial or financial relationships that could be construed as a potential conflict of interest.

## Abbreviations

GC: Advanced gastric adenocarcinoma
SG: Superficial gastritis
MetaPhlAn: Metagenomic phylogenetic analysis
HUMAnN: The HMP Unified Metabolic Analysis Network
LEfSe: Linear discriminant analysis effect size
BMI: Body mass index
PCOA: Principal coordinate analysis
PERMANOVA: Non-parametric permutational multivariate analysis of variance
LDA: Linear discriminant analysis
LPS: Lipopolysaccharide
FDR: False discovery rate
SCFAs: Short chain fatty acids
TCA: tricarboxylic acid.
